# Behavioural manipulation of insect hosts by *Baculoviridae* as a process of niche construction

**DOI:** 10.1186/1471-2148-13-170

**Published:** 2013-08-16

**Authors:** Steven Hamblin, Mark M Tanaka

**Affiliations:** 1 School of Biotechnology and Biomolecular Sciences and Evolution & Ecology Research Centre, The University of New South Wales, Kensington, NSW, Australia

## Abstract

**Background:**

Niche construction has received increasing attention in recent years as a vital force in evolution and examples of niche construction have been identified in a wide variety of taxa, but viruses are conspicuously absent. In this study we explore how niche construction can lead to viruses engineering their hosts (including behavioural manipulation) with feedback on selective pressures for viral transmission and virulence. To illustrate this concept we focus on *Baculoviridae*, a family of invertebrate viruses that have evolved to modify the feeding behaviour of their lepidopteran hosts and liquefy their cadavers as part of the course of infection.

**Results:**

We present a mathematical model showing how niche construction leads to feedback from the behavioural manipulation to the liquefaction of the host, linking the evolution of both of these traits, and show how this association arises from the action of niche construction. Model results show that niche construction is plausible in this system and delineates the conditions under which niche construction will occur. Niche construction in this system is also shown to be sensitive to parameter values that reflect ecological forces.

**Conclusions:**

Our model demonstrates that niche construction can be a potent force in viral evolution and can lead to the acquisition and maintenance of the behavioural manipulation and liquefaction traits in *Baculoviridae* via the niche constructing effects on the host. These results show the potential for niche construction theory to provide new insights into viral evolution.

## Background

Niche construction, defined as the “process whereby organisms, through their metabolism, their activities, and their choices, modify their own and/or each other’s niches” [1], has received growing attention as a potentially potent force in evolution. This act of modifying niches changes biotic and abiotic sources of selection in the organisms’s environment, introducing feedback into the evolutionary process as the effects of niche construction modify an organism’s genetic and ecological inheritance [2,3]. Examples of niche construction have been identified in all taxonomic groups, but little attention has been paid to viruses. As obligate intracellular parasites, viruses necessarily have ongoing (and often antagonistic) interactions with their hosts; by altering their hosts they are altering their own selective environments. When these interactions modify the host in a way that has lasting effects on viral selection pressure, niche construction will be in operation.

In a fashion similar to other parasites [4], host manipulations that create niche construction effects for viruses may occur at multiple levels of the host organisms. For example, at the level of the host’s physiology, viruses such as norovirus cause fever, diarrhoea, and vomiting. These can drive niche construction because of their direct effect on the host (e.g. transmission via viral shedding in diarrhoea), and there may be additional effects from the host’s response to these changes. Adelman and Martin [5] describe the behavioural changes in the host from fever and other symptoms as an ‘emergency life-history stage in which organisms prioritise recovery from infection’; the host response, such as lethargy or anorexia, may modify the virus’ own selective pressures. Viruses also directly cause gross changes in host behaviour, such as induction of pathological aggression in rabies, or reports of prolonged feeding by mosquitoes infected with dengue virus [6]. Viruses even have lasting effects on the environment beyond the host, as when marine viruses affect oceanic participation in carbon cycling [7]. Of course, this process of modifying the host may also change the host’s selective pressures; beyond the response of the immune system, the host may be under selection to counter the effects of the physiological or behavioural manipulations of the virus. Our understanding of viral evolution will be enhanced by identifying and examining traits that are coupled in this way, especially traits through which niche construction generates feedback into viral life history properties. In addition, the study of niche construction as an evolutionary process will be enhanced by new avenues for empirical tests in viruses. For example, we might compare populations of viruses in which the hypothesised niche construction pathway (such as a host manipulation enhancing transmission) is operating with populations in which this pathway has been experimentally negated to compare fitness or the evolution of viral traits [3]. With their short generation time and quick rates of evolution, viruses are attractive targets for empirical tests of niche construction theory.

In this study, we illustrate the operation of viral niche construction with an example drawn from the family of invertebrate viruses known as *Baculoviridae*. Baculoviruses are rod-shaped, enveloped dsDNA viruses with large genomes that range in size from 90 kb to 180 kb [8]. Two major baculovirus morphologies are distinguished, the nucleopolyhedroviruses (NPVs) and the granuloviruses (GVs) based on the contents of their occlusion bodies (OBs, the proteinaceous structures that contain the virions); NPVs have multiple virions per OB, while GVs typically have only one. NPVs are further subdivided into single or multiple NPVs (SNPV / MNPV) based on the contents of their virions, which can hold a single or multiple nucleocapsid. Baculoviruses infect arthropod hosts and have been reported worldwide from over 600 host species, primarily from the order Lepidoptera though they have been also identified in Diptera, Hymenoptera, and Decapoda as well [8]. This host specificity forms the basis for the currently accepted classification of the family *Baculoviridae* into four genera [9]: *Alphabaculovirus*, lepidopteran-specific NPVs, *Betabaculovirus*, lepidopteran-specific GVs, *Gammabaculovirus*, the hymenopteran-specific NPVs, and *Deltabaculovirus*, the dipteran-specific NPVs. Baculoviruses are evolutionarily old, with molecular estimates placing their origin around 310 million years ago during the Carboniferous period of the Paleozoic era [10].

In many lepidopteran host species it has been known since at least the 19th century that baculoviruses induce a behavioural modification in their hosts which results in forced climbing behaviour (referred to as “Wipfelkrankheit” or tree-top disease; Hoffman, cited by Goulson [11]). As part of their pathology, baculoviruses are also known to liquefy their hosts after death, allowing occluded virions to burst and enhance their dispersal [12]. Mechanistically, a small set of viral genes are known to be directly involved in these actions. Recent work has shown [13] that compared to controls, deleting the viral gene ecdysteroid UDP-glocosyltransferase(*egt*) in LdMNPV (host: *Lymantra dispar*) will disrupt the climbing behaviour and reinstating the gene also restores the climbing behaviour. Other genes, such as the viral gene for the baculovirus protein tyrosine phosphatase (*ptp*) of BmNPV (host: *Bombyx mori*), are occasionally implicated in the behavioural manipulation as well [14]. Important viral genes for liquefaction are chitinase and cathepsin (e.g. [15,16]), though other candidates may also be involved [17]. Genes such as *egt*, chitinase, and cathepsin are not necessarily the only causes for these traits, and given the constraints on viral genomes it is unsurprising that they may also have other functions (e.g. egt and virulence: [18,19]).

Despite the advances in understanding the mechanistic basis for host manipulation and liquefaction, insight into the evolutionary consequences and origins of these baculovirus characteristics remains speculative. It is thought that tree-top disease combined with liquefaction enhances transmission of the virus to conspecifics by shedding virus directly into the path of feeding conspecifics (as the virus infects orally). However, this leaves open the question of how such a conjunction of behaviours could arise to begin with. As Hoover et al. [13] note about *L. dispar*, in the absence of the virus caterpillars will hide in bark crevices or climb down the tree during the day to avoid predation from birds; this suggests that climbing behaviours without liquefaction would harm the transmission of the virus. Similarly, liquefaction of the host may have a much greater effect on transmission if it occurs at the top of the host plant / tree, as the virus is spread into the path of feeding conspecific by mechanical factors such as wind and rain. In contrast, liquefaction at the bottom of the host vegetation or in crevices may lead to sequestration of the virus in the soil and bark reservoir that leads to next season’s infections but reduce transmission in the current season, as virus left unprotected will degrade quickly from UV exposure or be removed by other mechanical means [20]. Thus, each feature of baculovirus pathology may be slightly beneficial, neutral, or even deleterious on its own, but feedback from one feature to the other may link the evolution of the two together.

**Figure 1:**
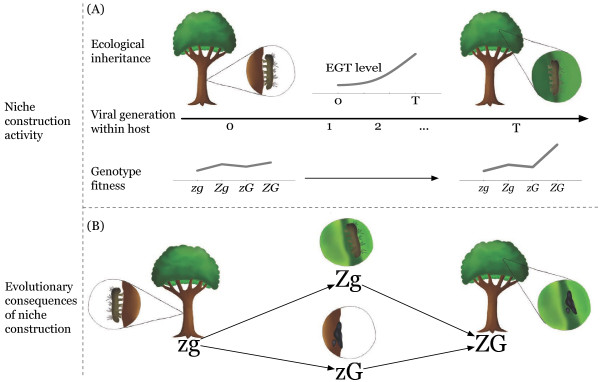
**Schematic representation of niche construction.** Conceptual representation of the action niche of construction in the baculovirus system. The build up of EGT within a lepidopteran host over successive viral generations **(A)** is an instance of ecological inheritance, which leads to a change in the host behaviour by time *T* (the ‘zombie’ trait of the virus; see Methods). This enhances the transmission of the virus if it liquefies the host (the ‘gooey’ trait). As described in ’Genetic structure’ below, we study four viral genotypes encoding these two traits: *zg* (non-zombie, non-gooey), *Zg* (zombie, non-gooey), *zG* (etc.), *ZG*; the genotype fitnesses in this figure are arbitrary chosen for illustrative purposes. In **(B)** we represent a potential pathway through genotype space for the virus over evolutionary time. **(A)** shows the change in fitness landscape as a result of the viral niche construction (i.e. its host manipulation), while **(B)** shows the evolutionary consequences of this niche construction activity for the virus.

In this study we aim to contribute to the broadening of the domain of niche construction to viral evolution. To do so, we apply a niche construction perspective to make a general evolutionary argument about the acquisition and maintenance of tree-top disease and liquefaction in baculoviruses infecting Lepidopteran hosts. We construct an epizootic model of baculovirus transmission to link viral fitness to genotypes with each combination of features and demonstrate that there are valid evolutionary paths from a hypothetical virus with neither feature to one such as LdMNPV with both (Figure 1).

## Methods

### Mathematical model of viral niche construction

#### ***Epizootic model***

To study the effects of baculovirus behavioural manipulation and liquefaction on viral fitness, we begin with a compartmental epizootic model similar to a Susceptible-Exposed-Infectious-Recovered (SEIR) model [21]. Our model tracks the status of the host population as it transitions through five states (c.f. [20,22]): susceptible individuals (*S*) that have not been exposed to the virus, exposed individuals (*E*) that have ingested the virus, intact cadavers (*C*) that spread the virus at a low rate, liquefied cadavers (*L*) that spread virus at a higher rate, and a reservoir (*V*) that harbours virus for the next epizootic outbreak (tracked as proportions, not absolute numbers of individuals). Epizootics are assumed to begin when some small proportion of the recently hatched larvae come in contact with virus from the previous season’s reservoir and become infected (*E*(0) ≈ 1.0 × 10^-5^). Susceptible individuals enter the exposed class by coming in contact with intact or liquefied cadavers, after which they die at some rate *ν*.

Intact cadavers infect susceptibles at a rate *β*_*C*_, are liquefied at a rate *λ*, and shed virus directly into the reservoir at a rate *θ*. The *egt* gene also increases the virion yield by deactivating moulting hormone and causing infected larvae to grow larger and die later than uninfected larvae. This potentially niche constructing effect can be incorporated into the transmission parameters *β*_*L*_ and *β*_*C*_, as an increased virion yield would increase transmission.

**Figure 2:**
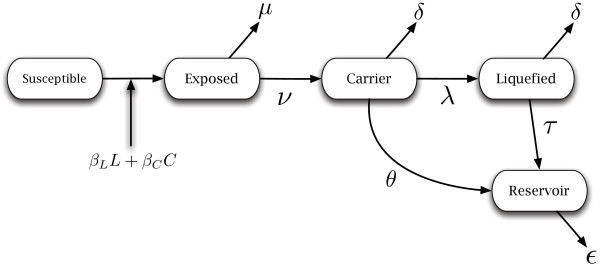
**Model diagram.** Model diagram. Susceptibles are infected at rates *β*_*C*_ and *β*_*L*_ from intact and liquefied cadavers respectively. Exposed hosts die from infection at rate *ν* and from natural causes or predation at rate *μ*. Intact cadavers are liquefied at rate *λ* and contribute to the reservoir at rate *θ*. Both intact and liquefied cadavers are removed by predators and other causes at the same rate *δ*. Liquefied cadavers contribute to the reservoir at rate *θ*, and virus in the reservoir degrades at rate *ε*. The parameters are summarised in Table 1.

As we are tracking the infection for a single season, we presume a host in which eggs overwinter and hatch in spring; thus, we neglect births in the model, and track only baseline mortality from natural causes and predation (*μ*). However, we do allow for excess mortality as a consequence of viral infection. For instance, exposed hosts suffer from reduced mobility and behavioural changes that may increase their chance of being predated (e.g. [13,23]) or cannibalised [24], and so this rate which we denote *δ* is higher as the disease progresses (*δ* > *μ*). Intact cadavers that die are liquefied internally until their cuticle bursts in a matter of hours; during this time, they can be removed by predation [12] at the same *δ* rate as intact cadavers, after which they transmit to susceptibles at a rate *β*_*L*_, and contribute to the reservoir at a rate *τ*. Virus that enters the reservoir degrades at some small rate *ε*. Note that this model includes a protected reservoir that will cause future outbreaks *sensu*[22] but, as shown by [25], simple environmental persistence of the virus could be used in the model instead with the same result. A flow diagram of this model is given in Figure 2. We focus on the dynamics of acute infection by baculovirus of a local population of larval hosts and consider long-term niche construction arising from these dynamics.

We model the movement of hosts between compartments as a set of ordinary differential equations as follows.

(1) $$\begin{array}{ll} \dot{S} & =-\beta_{L}\text{LS}-\beta_{C}\text{CS}\; \end{array}$$

(2) $$\begin{array}{ll} Ė & =\beta_{L}\text{LS}+\beta_{C}\text{CS}-(\mu +\nu )E\; \end{array}$$

(3) $$\begin{array}{ll} Ċ & =\mathrm{\nu E}-(\lambda +\theta +\delta )C\; \end{array}$$

(4) $$\begin{array}{ll} \dot{L} & =\mathrm{\lambda C}-(\tau +\delta )L\; \end{array}$$

(5) $$\begin{array}{ll} \dot{V} & =\mathrm{\theta C}+\mathrm{\tau L}-\mathrm{\varepsilon V.}\; \end{array}$$

The parameters for this model and their meanings can be found in Table 1 (parameters values are discussed below). We follow [21] and [26] (see also [27]) to calculate the basic reproduction number *R*_0_ of this model using the next generation matrix, as follows.

**Table 1:** Parameter values

**Parameter**	**Meaning**
*β*_*L*_	Transmission between susceptibles and liquefied cadavers.
*β*_*C*_	Transmission between susceptibles and intact cadavers.
*μ*	Baseline death rate for susceptible and exposed
	(but asymptomatic) hosts.
*ν*	Rate of conversion from exposed to intact cadaver states.
*δ*	Clearance rate for intact and liquefied cadavers.
	Includes predation and cannibalism that does not transmit
	infection.
*λ*	Rate of conversion of intact to liquefied cadavers.
*τ*	Rate at which virus shed from liquefied cadavers enters
	the reservoir.
*θ*	Rate of virus shedding into the reservoir from intact
	cadavers.
*ε*	Clearance rate of virions from the reservoir by
	environmental factors (UV degradation, wind, rain, etc).

We order the compartments of the model as (*E*,*C*,*L*,*S*,*V*) so that the first *m* = 3 contain infected individuals. Let *x* = (*x*_1_,…,*x*_*n*_)^*t*^,*x*_*i*_ ≥ 0 be the corresponding number of individuals in each disease compartment. Let $F_{i}(x)$ be the rate of new infections appearing in compartment *i*, and $V_{i}(x)$ be the net transfer of individuals into compartment *i*. The difference $F_{i}(x)-V_{i}(x)$ gives the rate of change of *x*_*i*_.

In our model,

(6) $$\begin{array}{ll} \{F_{i}\}\;=\;\left(\;\;\begin{array}{c} \beta_{L}\text{LS}+\beta_{C}\text{CS}\\ 0\\ 0\\ 0\\ 0 \end{array}\;\right)\;\text{and}\;\{V_{i}\}\;=\;\left(\;\;\begin{array}{c} (\mu +\nu )E\\ -\mathrm{\nu E}\;+\;(\lambda \;+\;\theta \;+\;\delta )C\\ -\mathrm{\lambda C}\;+\;(\tau \;+\;\delta )L\\ \beta_{L}\text{LS}\;+\;\beta_{C}\text{CS}\\ -\mathrm{\theta C}-\mathrm{\tau L}+\mathrm{\varepsilon V} \end{array}\;,\;\right)\;. & \end{array}$$

We form *m* × *m* matrices *F* and *V* whose elements are given by

(7) $$\begin{array}{ll} F_{i,j}=\left[\;\frac{\partial F_{i}(x_{0})}{\partial x_{j}}\;\right]\;\text{and}\;V_{i,j}=\left[\;\frac{\partial V_{i}(x_{0})}{\partial x_{j}}\;\right], & \end{array}$$

where *x*_0_ is the disease-free equilibrium (here, *x*_0_ = [0,0,0,1,0]), and *i*,*j* ≤ *m*.

Using equation (7), *F* and *V* are

$$\begin{array}{lll} F & =\left(\;\begin{array}{ccc} 0 & \beta_{C} & \beta_{L}\\ 0 & 0 & 0\\ 0 & 0 & 0 \end{array}\;\right)\; & \end{array}$$

and

$$\begin{array}{lll} V & =\left(\;\begin{array}{ccc} \mu +\nu & 0 & 0\\ -\nu & \lambda +\theta +\delta & 0\\ 0 & -\lambda & \tau +\delta \end{array}\;\right).\; & \end{array}$$

The basic reproduction number *R*_0_ of the model is the spectral radius of the next generation matrix which is given by *FV*^-1^, which simplifies to

(8) $$\begin{array}{ll} R_{0} & =\frac{\beta_{C}\nu (\delta +\tau )+\beta_{L}\nu \lambda }{(\delta +\theta +\lambda )(\mu +\nu )(\delta +\tau ).}\; \end{array}$$

We can clarify the contribution of each model component by rewriting this expression as

(9) $$\begin{array}{ll} R_{0} & \;=\;\frac{\beta_{C}}{\delta +\theta +\lambda }\left(\;\frac{\nu }{\nu +\mu }\;\right)+\frac{\beta_{L}}{\delta +\tau }\left(\;\frac{\nu }{\nu +\mu }\;\right)\left(\;\frac{\lambda }{\delta +\theta +\lambda }\;\right)\; \end{array}$$

The first term above gives the number of new infections produced by an intact cadaver, over (*δ* + *θ* + *λ*)^-1^ time units. It includes the factor *ν*/(*ν* + *μ*) which is the proportion of exposed individuals that die. The second component corresponds to the contribution of the liquefied state and includes both the proportion entering the cadaver state from the exposed state and the proportion entering the liquefied state from the cadaver state, given by *λ*/(*λ* + *δ* + *θ*).

#### ***Genetic structure***

To trace the selective effects of the behavioural manipulation and liquefaction features of baculovirus, we define four genotypes from two hypothetical loci with two alleles each. For the purposes of our model we adopt a simplified view of *egt* and chitinase / cathepsin as being responsible for the host manipulation and liquefaction traits respectively (Figureῠ1); further, we adopt non-specific names for these traits to highlight the general nature of our evolutionary argument which shows the niche constructive pathway from a historical but unknown protovirus with neither trait to a modern virus with both. *Z* and *z* are zombie and non-zombie alleles, corresponding to the presence or absence of the behavioural manipulation driven by the *egt* (and *ptp*) gene. *G* and *g* are gooey and non-gooey alleles, corresponding to the presence of the liquefying genes cathepsin and chitinase. This gives us *zg, Zg, zG, ZG* as the set of genotypes in the system, each with its own basic reproduction number *R*_0,*i*_ where *i* denotes the genotype (with *i* ∈ {*zg*,*Zg*,*zG*,*ZG*}).

As shown in Table 2, we define the effect of the genotypes by their effect on viral life history traits via model parameters, with each genotype having its own value of four critical parameters indexed by genotype: namely, for the *i*^*th*^ genotype, *β*_*Li*_, *τ*_*i*_, *λ*_*i*_, and *θ*_*i*_. Note that we replace the generic symbols introduced in the section above describing the ecology of disease spread with new notation given in Table 2, which depend on genotype. Here we introduce a new parameter, *γ*, which represents the advantage or disadvantage to each mode of transmission (horizontal or via the reservoir in next season). Relative to the *zg* genotype *ZG* increases horizontal transmission by liquefying ‘tree-top’ (plant-top, etc) larvae and increasing spread of occluded virus over foliage that conspecific larvae will eat (increasing *β*_*L*,*ZG*_). However, this reduces contribution to the reservoir (lowering *τ*_*ZG*_) because exposed virus will degrade quickly, especially as a result of UV light ([28], p.364-5); in the past, this fact limited the potential use of baculoviruses as insecticides. We assume that the reverse happens to the *zG* genotype, in which insects die in a natural position closer to the soil or understory and then liquefy to spread virus to the reservoir (soil, understory, tree bark, etc). Occluded NPVs in forest settings have been shown to persist as long as 41 years after a natural outbreak [29]. Thus, *γ* decreases *β*_*L*,*zG*_ and reduces *τ*_*zG*_ relative to the *zg* genotype. Since they do not liquefy their host (*λ*_*zg*_, *λ*_*Zg*_ = 0), it is assumed that the *Zg* and *zg* genotypes are neutral with respect to transmission of the virus (relative to the protovirus *zg*). However, the zombie allele would still modify contribution to the reservoir by the same argument as above, so we can represent this by modifying the values of *θ*_*i*_; we represent this as a low value of *θ*_*i*_, *θ*_*l*_ for the *Z* allele and a high value *θ*_*h*_ for the *z* allele (*θ*_*h*_ > *θ*_*l*_).

**Table 2:** Genotypes

***i***	***β***_***L*,*i***_	***τ***_***i***_	***λ***_***i***_	***θ***_***i***_
*ZG*	*β*_*L*_(1 + *γ*)	*τ*(1 - *γ*)	*λ*	*θ*_*l*_
*Zg*	*β*_*L*_	*τ*	0	*θ*_*l*_
*zG*	*β*_*L*_(1- *γ*)	*τ*(1 + *γ*)	*λ*	*θ*_*h*_
*zg*	*β*_*L*_	*τ*	0	*θ*_*h*_

Genotypes and their effect on the model parameters.

Based on the principle of competitive exclusion in pathogen virulence [30], we assume that evolution follows a path in which viruses of higher *R*_0,*i*_ invade and replace those of lower *R*_0,*i*_; this approach is widely used in evolutionary epidemiology, as noted by [31]. Competitive exclusion also allows us to ignore the dynamics of the reservoir, as we assume that the dominant genotype will also be dominant in the reservoir, and the seasonal dynamics of baculovirus have already been modelled (e.g. [22]). Thus, when the *ZG* genotype has the highest fitness value, we conclude that the trajectory through genotype space will proceed according to the changed selective pressures imposed by niche construction (Figure 1). If *ZG* does not have the highest fitness in some region of the parameter space, we conclude that niche construction does not occur. Thus, using values of the basic reproduction number *R*_0,*i*_ as a function of genotype, we explored the potential for niche construction in parameter space by defining fitness orderings by genotype. Plotting viral fitness over *γ* allows us to rank the value of *R*_0,*i*_ for each genotype at a given set of parameter values in order from lowest to highest, yielding a fitness ordering to which we assign a value and a colour (Table 3).

**Table 3:** Color mapping

**Fitness ordering**	**Color**
Any in which *ZG* doesn’t have the highest fitness.	Purple
*zG* < *Zg* < *zg* < *ZG*	Blue
*Zg* < *zG* < *zg* < *ZG*	Teal
*Zg* < *zg* < *zG* < *ZG*	Green
*zG* < *zg* < *Zg* < *ZG*	Yellow
*zg* < *Zg* < *zG* < *ZG*	Orange
*zg* < *zG* < *Zg* < *ZG*	Red

Mapping of the niche construction evolutionary pathways to colors.

#### ***Parameter values***

For numerical analyses of the model, we set the baseline parameters as described in Table 1 as follows (all units are rates per individual per day). Parameters *β*_*L*_ and *β*_*C*_ were set to 1 and 0.5 per day respectively, to provide a large transmission advantage to liquefied cadavers, following baculovirus pathology and ecology [12]. Contribution to the reservoir, *τ* (set to 0.2) and *θ* (set to 0.25), is expected to be low (conditional on where the virus is shed). For the remainder of this study we neglect mortality to susceptible and exposed hosts (*μ*) and clearance from the reservoir (*ε*) as being unimportant for the niche construction aspect of the model and so set them to zero, but deaths from predation may have an effect on viral fitness and so we set a low value of *δ* = 0.05 as a baseline. Finally, we take the average time from exposure to death as being 5–10 days (as is typical for fourth- or fifth-instar infections [23]), and so set the rate of disease progression from exposed to intact cadaver (*ν*) to 1/7 = 0.14. In exploring the effect of model parameters on niche construction, we varied *β*_*L*_ from 0.5 to 2, *β*_*C*_ from 0.1 to 1, *θ*_*l*_ from 0.01 to 0.5, *λ* from 0.1 to 2, *δ* from 0 to 0.5, and *τ* from 0.01 to 0.5.

#### ***Model analysis***

We numerically solved equations (1)-(5) using Mathematica [32]. Initial conditions were set to *C*(0)→1×10^-8^,*E*(0) → 0,*L*( 0) → 0,*V*( 0) → 0, and *S* (0) →1 -(*E*(0) + *C*(0) + *L*(0) + *V*(0)). Calculation and plotting of genotype fitness and fitness orderings over parameter space was done using R [33].

## Results

### Model results

**Figure 3:**
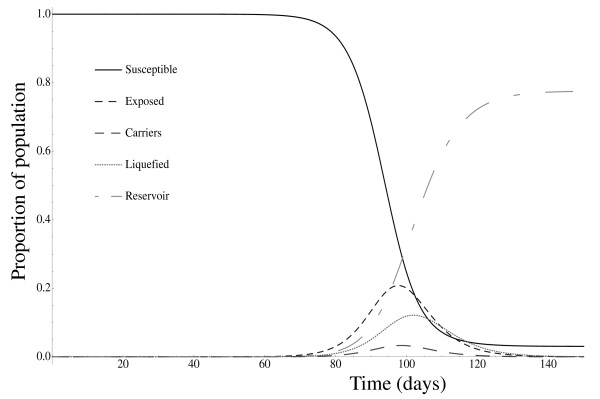
**Epizootic dynamics.** Epizootic dynamics over time, showing the progress of the infection in a susceptible population that has been exposed to a single infectious individual. For this figure, *β*_*L*_ = 1, *β*_*C*_ = 0.5, *τ* = 0.2, *θ* = 0.25, *ν* = 0.14, *δ* = 0.05, *μ* = 0, *ε* = 0.

Figure 3 shows the course of infection under the model using the parameters values given in Section ‘Parameter values’. These dynamics are qualitatively in line with known properties of baculovirus infections of species such as *L. dispar* (e.g. [34,35]). Larvae of *L. dispar* hatch in early spring and proceed through five or six instars of roughly two weeks each [36].

**Figure 4:**
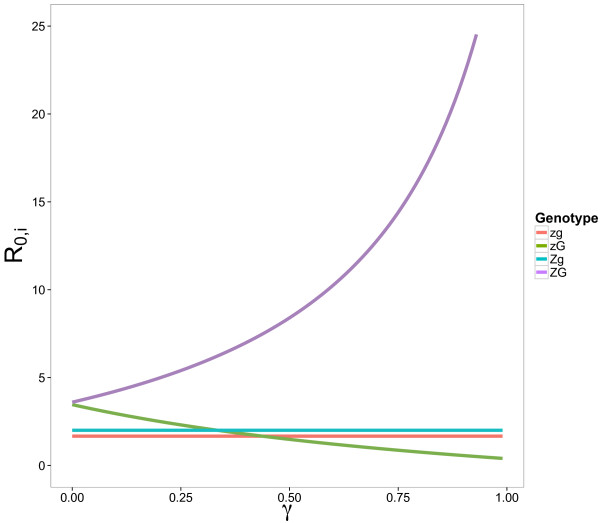
**Viral fitness as a function of*γ*.** Plot of viral fitness (measured by *R*_0_) as a function of the advantage parameter *γ*. For this figure, *β*_*L*_ =1 , *β*_*C*_ = 0.5, *τ* = 0.2, *θ* = 0.25, *ν* = 0.14, *δ* = 0.05, *μ* = 0, *ε* = 0. Slicing through the graph vertically at a fixed value of *γ* allows us define the fitness orderings in Table 3.

Viral fitness (*R*_0_) as a function of genotype and the advantage parameter *γ* is shown in Figure 4. For this combination of parameters, low values of *γ* (less than 0.3) lead to evolution proceeding from the protovirus with neither trait (*zg*) and acquiring either trait first before arriving at the zombie-gooey genotype *ZG* via the niche constructive feedback on the virus’s selective pressures. At higher values of *γ* (greater than 0.5), we see a fitness ordering that only allows niche construction to proceed by acquiring the zombie trait first (*ZG*).

**Figure 5:**
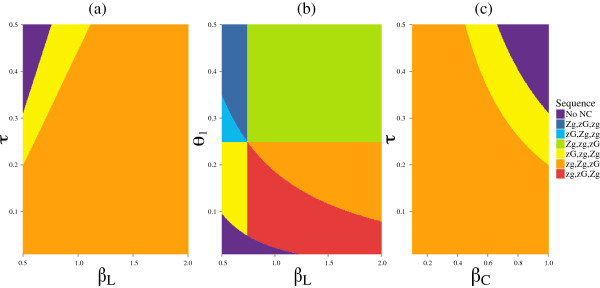
**Fitness orderings.** Fitness orderings showing the plausibility of niche construction on the zombie and gooey traits, across parameter space. Plots are for *γ* values of 0.1 (panel **a**), 0.3 **(b)**, and 0.1 **(c)** ; if not varying along the x- or y-axis, parameters values are *β*_*L*_ = 1, *β*_*C*_ = 0.5, *τ* = 0.2, *θ* = 0.25, *ν* = 0.14, *δ* = 0.05, *μ* = 0, *ε* = 0.

Figure 5 shows the plausibility of niche construction under various model parameters. Our results show that niche construction is plausible in this system for a wide range of parameters; we also see that some combinations of parameters do not allow niche construction. The rates of transmission (*β*_*L*_, *β*_*C*_), combined with the rate at which the virus is shed into the reservoir (*τ*, *θ*) from either source affect the sequence of trait acquisition that is most profitable for the virus, such that low / high transmission from liquefied and intact cadavers respectively restricts the virus to acquiring the zombie trait to increase its fitness when the contribution to the reservoir is high (Figure 5(a,c)). The value of *θ*_*l*_ shows the relative contribution to the reservoir from the *Z* allele compared to the *z* allele. Because *θ*_*h*_ was set to 0.25 (Table 1), the vertical axis of Figure 5(b) is the difference between the *Z* and *z* alleles. Thus, when *θ*_*l*_ goes above 0.25, the effect of the zombie trait inverts. The interplay between these parameters defines regions in which the transmission from liquefied cadavers shows a trade-off with the contribution to the reservoir made by zombie caterpillars. When *θ*_*l*_ is low (large effect of *Z*) and transmission is low, no niche construction occurs, but high transmission rescues the niche constructive pathway. When the effect of *Z* inverts, high rates of transmission still allow niche construction to acquire both traits (green region), but only through the gooey trait. And at low rates of transmission, niche construction may maintain the two traits but is unlikely to lead to their acquisition (blue region), as going from *zg* to *ZG* now requires acquiring both traits simultaneously.

## Discussion

In this study we have explored the potential for niche construction to play a role in the acquisition and maintenance of a significant manipulation of host behaviour by members of the *Baculoviridae* family. Results from our modelling suggest that the feedback from manipulating the host is sufficient to acquire and maintain both the behavioural manipulation (zombie) and liquefaction (gooey) traits. Niche construction provides a strong explanation for the acquisition of these traits by virus, because the ecological inheritance from manipulating the host via the expression of the *egt* gene changes the selective pressures over successive viral generations until liquefaction changes from neutral or slightly advantageous to highly advantageous (Figure 1A). The resulting fitness landscape, which includes epistatic interaction between the loci, is an effect of the process of niche construction that we model here. This process affects the evolution of the virus over subsequent transmission events and seasonal infections, since the virus receives greater fitness benefits as it travels through genotype space (Figure 1B) by acquiring both traits (and possibly fine-tuning their expression). It is also worth noting that the system we are modelling is an example of the *inceptive perturbation* category of niche construction described in Table 3.1 of [37], wherein “organisms initiate a change in their selective environment by physically modifying their surroundings”. This category also applies to the possible niche construction effect of *egt* in which the deactivation of moulting in the larvae changes their growth pattern and lifespan and thus modifies the occlusion body yield of the cadaver.

Our model results are sensitive to parameter values that reflect ecological forces. For instance, differences in host ecology across species such as the choice of host plant and density of hosts per plant could affect transmission to conspecifics or increase contribution to the reservoir, which would change the predictions of our model as to whether niche construction would favour *ZG* genotypes or the way in which they might have been acquired. Examples of differing host ecology abound; for instance, caterpillars of *Lymantria dispar* feed on leaves near the tops of trees, providing boosts to horizontal transmission (increasing *β*_*L*,*ZG*_) and favouring *ZG*. On the other hand, larvae of crop pests like *Anticarsia gemmatalis* (velvetbean caterpillars) that feed on soybean and other legume plants have a dispersed spatial distribution [38] and are active enough to jump off the plant if disturbed. This may not favour the *G* allele, which may account for the lack of liquefaction in this NPV [39], and whether the virus manipulates its host is unknown.

We argue that niche construction provides a more complete view of the benefits of host manipulation to viruses by providing a clear link between traits that, together, enhance fitness through the action on the host. Alternative explanations, such as standard natural selection with epistasis between the zombie and gooey traits, are not sufficient because they do not account for the fitnesses changing over time in this system; the zombie trait creates an ecological inheritance that builds up and cannot affect the fitness of the gooey trait until the behavioural manipulation has run its course. For *Baculoviridae*, this allows us to explain why the virus would manipulate its host’s behaviour in such a dramatic fashion; the resulting change in the host creates conditions that modify the effect of the liquefaction trait on viral fitness and couple the two traits together. The niche construction explanation for viral manipulation of hosts can also provide testable predictions for empirical work; our model predicts that, when niche construction is favoured, disrupting the link between between the zombie and gooey traits by blocking the manipulation inspired by genes like *egt* should render both (nearly) neutral and thus subject to silencing or removal. This may also help to explain why in vitro cultures of NPVs often lose their *egt* genes quickly and show much greater levels of virulence [18,19], and might provide insights into methods for enhancing the effectiveness of baculoviruses as biocontrol agents for their lepidopteran hosts, which are often agricultural pests. We note that the focus of much parasitological literature on the evolution of host manipulation by parasites has been on extended phenotype theory as ‘a way of viewing the facts’ [40], though the structure of our model does not depend on the details of the distinction between extended phenotype and niche construction [41,42].

Empirically, the family Baculoviridae has been extensively studied on a molecular level, both for its use as a vector for protein expression and as a possible biopesticide. In addition to this, sufficient basic research on the properties of its pathology and ecological inheritance is available to make studying niche construction in this system possible; all that remains is to put the pieces together appropriately. For example, recombinant strains with *egt* and chitinase/cathepsin deletions have been created (separately) for LdMNPV, and the effects of the deletion on host behaviour / transmission studied (e.g. egt: [13], and chitinase: [43]). The next step is to disrupt the niche-constructed pathway in an experimental setting. One possible experimental design would be to create the four genotypes in our model (*zg,Zg,zG,ZG*) and compare their effects on transmission in the lab and in the field, using the methodology of D’Amico et al. [43] (confinement of larval treatment conditions to mesh bags in a naturalistic setting) to measure transmission. Replicating the fitness orderings from our model would provide support to our predictions. It should also be possible to use an ecological manipulation to test this model. If larvae infected with *ZG* (wildtype) viruses are prevented from experiencing the behavioral effects of tree-top disease (i.e. climbing to the top of the host plant), then over multiple generations we expect rapid loss of *egt* and (or) chitinase or cathepsin in treatment strains but not control strains that are free to manipulate the host.

With over 600 viruses described in the literature [44] and mixed success implementing baculoviruses as pesticides to date [45], finding hosts with ecology favouring the niche constructive pathway we have identified in this study may aid in designing more effective baculovirus biocontrol agents. For instance, it may be possible to identify which hosts will benefit from increased viral transmission via this niche constructed pathway and target the engineering of baculovirus biopesticides accordingly. It may also be possible to manipulate the environment of the host to work with the virus. For example, if the effect of zombie gooey phenotypes is enhanced by a clustered spatial distribution of host plants, baculoviruses targeted at crop pests may be improved by modifying the planting regime to match. Further basic research on the effects of niche construction in this viral family thus holds the potential to benefit our understanding of viral evolution as well as enhance practical applications of *Baculoviridae*.

## Conclusions

Niche construction provides a new lens through which to study viral manipulations of the host. The analysis of the mathematical model developed in this study has shown that niche construction provides a plausible explanation for the behavioural manipulation and liquefaction of Lepidopteran hosts by baculoviruses. Understanding the evolutionary forces behind these manipulations provides not only new insights into viral evolution, but can form the basis for new empirical work on baculoviruses and research on the use of these viruses as biocontrol agents.

## Competing interests

Both authors declare that they have no competing interests.

## Authors’ contributions

SH and MT conceived and designed the study, SH produced the data and analysed it with MT. SH and MT wrote the manuscript. Both authors read and approved the final manuscript.

## References

[B1] Laland KN Sterelny K Seven reasons (not) to neglect niche construction Evolution 2006 60 9 1751 1762 17089961

[B2] Kerr B Schwilk DW Bergman A Feldman MW Rekindling an old flame: a haploid model for the evolution and impact of flammability in resprouting plants Evol Ecol Res 1999 1 807 833

[B3] Day RL Laland KN Odling-Smee J Rethinking adaptation: the niche-construction perpsective Perspect Biol Med 2003 46 80 95 10.1353/pbm.2003.0003 12582272

[B4] Hughes DJ Brodeur J Thomas F Host Manipulation by Parasites 2012 Oxfrod: Oxford University Press

[B5] Adelman JS Martin LB Vertebrate sickness behaviors: adaptive and integrated neuroendocrine immune responses Integr Comp Biol 2009 49 3 202 214 10.1093/icb/icp028 21665814

[B6] Platt KB Linthicum KJ Myint KSA Innis BL Lerdthusnee K Vaughn DW Impact of dengue virus infection on feeding behavior of Aedes aegypti Am J Trop Med Hygiene 1997 57 2 119 125 10.4269/ajtmh.1997.57.119 9288801

[B7] Suttle CA Viruses in the sea Nature 2005 437 356 361 10.1038/nature04160 16163346

[B8] Herniou EA Olszewski JA Cory JS O’Reilly DR The genome sequence and evolution of baculoviruses Ann Rev Entomol 2003 48 211 34 10.1146/annurev.ento.48.091801.112756 12414741

[B9] Jehle JA Blissard GW Bonning BC Cory JS Herniou EA Rohrmann GF Theilmann DA Thiem SM Vlak JM On the classification and nomenclature of baculoviruses: a proposal for revision Arch Virol 2006 151 7 1257 1266 10.1007/s00705-006-0763-6 16648963

[B10] Thézé J Bézier A Periquet G Drezen J Herniou EA Paleozoic origin of insect large dsDNA viruses Proc Natl Acad Sci 2011 108 38 15931 15935 [http://www.pnas.org/content/108/38/15931] 10.1073/pnas.1105580108 21911395 PMC3179036

[B11] Goulson D Wipfelkrankheit: modification of host behaviour during baculoviral infection Oecologia 1997 109 219 228 10.1007/s004420050076 28307172

[B12] Cory JS Myers JH The ecology and evolution of insect baculoviruses Ann Rev Ecol Evol Systematics 2003 34 239 272 10.1146/annurev.ecolsys.34.011802.132402

[B13] Hoover K Grove M Gardner M Hughes DP McNeil J Slavicek J A gene for an extended phenotype Science 2011 333 1401 10.1126/science.1209199 21903803

[B14] Katsuma S Koyano Y Kang W Kokusho R Kamita SG Shimada T The baculovirus uses a captured host phosphatase to induce enhanced locomotory activity in host caterpillars PLoS Pathog 2012 8 4 e1002644 10.1371/journal.ppat.1002644 22496662 PMC3320614

[B15] Hawtin RE Zarkowska T Arnold K Thomas CJ Gooday GW King LA Kuzio JA Possee RD Liquefaction of Autographa california Nucleopolyhedrovirus-infected insects is dependent on the integrity of virus-encoded chitinase and cathepsin genes Virology 1997 238 243 253 10.1006/viro.1997.8816 9400597

[B16] Wang Y Choi JY Roh JY Liu Q Tao XY Park JB Kim JS Je YH Genomic sequence analysis of granulovirus isolated from the tobacco cutworm, Spodoptera litura PLoS ONE 2011 6 11 e28163 10.1371/journal.pone.0028163 22132235 PMC3223241

[B17] Katsuma S Noguchi Y Zhou CLE Kobayashi M Maeda S Characterization of the 25K FP gene of the baculovirus Bombyx mori nucleopolyhedrovirus: implications for post-mortem host degredation J Gen Virol 1999 80 783 791 10092020 10.1099/0022-1317-80-3-783

[B18] O’Reilly DR Miller LK Improvement of a baculovirus pesticide by deletion of the egt gene Nat Biotechnol 1991 9 1086 1089 10.1038/nbt1191-1086

[B19] Erlandson MA Genetic variation in field populations of baculoviruses: mechanisms for generating variation and its potential role in baculovirus epizootiology Virol Sinica 2009 24 4 458 469

[B20] Cory JS Hails RS Sait SM Miller LK, Miller LK Baculovirus ecology The Baculoviruses 1997 New York: Plenum Press 301 339

[B21] Diekmann O Heesterbeek JAP Mathematical Epidemiology of Infectious Diseases: Model Building, Analysis, and Interpretation. Wiley Series in Mathematical and Computational Biology 2000 Chichester: John Wiley and Sons, Inc.

[B22] Hochberg ME The potential role of pathogens in biological control Nature 1989 337 262 265 10.1038/337262a0 2911366

[B23] Federici BA Miller LK Baculovirus pathogenesis The Baculoviruses 1997 New York: Plenum Press 33 59

[B24] Elvira S Williams T Caballero P Juvenile hormone analog technology: effects on larval cannibalism and the production of Spodoptera exigua (Lepidoptera: Noctuidae) nucleopolyhedrovirus Biol Microb Control 2010 103 3 577 582 10.1603/ec09325 20568601

[B25] Fuller E Elderd BD Dwyer G Pathogen persistence in the environment and insect-baculovirus interactions: disease-density thresholds, epidemic burnout, and insect outbreaks Am Nat 2012 179 3 E70—E96 22322229 10.1086/664488 PMC3814039

[B26] van den Driessche P Watmough J Reproduction numbers and sub-threshold endemic equilibria for compartmental models of disease transmission Math Biosci 2002 180 29 48 10.1016/S0025-5564(02)00108-6 12387915

[B27] Hurford A Cownden D Day T Next-generation tools for evolutionary invasion analyses J R Soc Interface 2010 7 561 571 10.1098/rsif.2009.0448 19955121 PMC2842787

[B28] Black BC Brennan LA Dierks PM Gard IE Miller LK, Miller LK Commercialization of baculoviral insecticides The Baculoviruses 1997 New York: Plenum Press 341 387

[B29] Fuxa JR Richter AR Quantification of soil-to-plant transport of recombinant nucleopolyhedrovirus: effects of soil type and moister, air currents, and precipitation Appl Environ Microbiol 2001 67 11 5166 10.1128/AEM.67.11.5166-5170.2001 11679341 PMC93286

[B30] Bremermann HJ Thieme HR A competitive exclusion principle for pathogen virulence J Math Biol 1989 27 179 190 10.1007/BF00276102 2723551

[B31] Day T Proulx SR A general theory for the evolutionary dynamics of virulence Am Nat 2004 163 4 E40—E63 15122509 10.1086/382548

[B32] Wolfram Research, Inc Mathematica Edition: Version 8.0 2010 Champaign: Wolfram Research, Inc.

[B33] R Development Core Team R: A Language and Environment for Statistical Computing 2007 Vienna: R Foundation for Statistical Computing

[B34] Doane CC Primary pathogens and their role in the development of epizootic in the gypsy moth J Invert Pathol 1970 15 21 33 10.1016/0022-2011(70)90094-7

[B35] Woods SA Elkinton JS Bimodal patterns of mortality from nuclear polyhedrosis virus in gypsy moth (Lymantria dispar) populations J Invert Pathol 1987 50 151 157 10.1016/0022-2011(87)90115-7

[B36] Elkinton JS Liebhold AM Population dynamics of gypsy moth in North America Ann Rev Entomol 1990 35 571 596 10.1146/annurev.en.35.010190.003035

[B37] Odling-Smee FJ Laland KN Feldman MW Niche Construction: The Neglected Process in Evolution. Monographs in Population Biology 2003 Princeton: Princeton University Press

[B38] Shepard M Carner GR Distribution of insects in soybean fields Can Entomologist 1976 108 7 767 771 10.4039/Ent108767-7

[B39] Slack JM Ribeiro BM de Souza ML The gp64 locus of Anticarsia gemmatalis multicapsid nucleopolyhedrovirus containsa 3’ repair exonuclease homolgoue and lacks v-cath and ChiA genes J Gen Virol 2004 85 211 219 10.1099/vir.0.19617-0 14718636

[B40] Lefèvre T Adamo SA Biron DG Missé D Hughes D Thomas F Invasion of the body snatchers: the diversity and evolution of manipulation strategies in host-paraiste interactions Adv Parasitol 2009 68 45 83 19289190 10.1016/S0065-308X(08)00603-9

[B41] Laland KN Extending the extended phenotype Biol Philos 2004 19 313 325

[B42] Dawkins R Extended phenotype - but not too extended. A reply to Laland, Turner, and Jablonka Biol Philos 2004 19 377 396

[B43] D’Amico V Slavicek J Podgwaite JD Webb R Fuester R Peiffer RA Deletion of v-chiA from a baculovirus reduces horizontal transmission in the field Appl Environ Microbiol 2013 79 13 4056 4064 10.1128/AEM.00152-13 23624474 PMC3697571

[B44] Herniou EA Jehle JA Baculovirus phylogeny and evolution Curr Drug Targets 2007 8 1043 1050 10.2174/138945007782151306 17979664

[B45] Moscardi F de Souza ML de Castro MEB Moscardi ML Szewczyk B Ahmad I, Ahmad F, Pichtel J Baculovirus pesticides: present state and future perspectives. Microbes and Microbial Technology: Agricultural and Environmental Applications, chapter 16 2011 New York: Springer 415 445

